# Educators’ perspectives of adopting virtual patient online learning tools to teach clinical reasoning in medical schools: a qualitative study

**DOI:** 10.1186/s12909-023-04422-x

**Published:** 2023-06-08

**Authors:** A.P Kassianos, R Plackett, M.A Kambouri, J Sheringham

**Affiliations:** 1 Department of Applied Health Research, University College London (UCL), 1-19 Torrington Place, London, WC1E 7HB UK; 2 Institute of Education, University College London (UCL), London, UK

**Keywords:** Simulation learning, Clinical reasoning, Adoption, Implementation framework, Online learning, Virtual patient, Medical education, Medical students

## Abstract

**Background:**

Learning tools using virtual patients can be used to teach clinical reasoning (CR) skills and overcome limitations of using face-to-face methods. However, the adoption of new tools is often challenging. The aim of this study was to explore UK medical educators’ perspectives of what influences the adoption of virtual patient learning tools to teach CR.

**Methods:**

A qualitative research study using semi-structured telephone interviews with medical educators in the UK with control over teaching materials of CR was conducted. The Consolidated Framework for Implementation Research (CFIR), commonly used in healthcare services implementation research was adapted to inform the analysis. Thematic analysis was used to analyse the data.

**Results:**

Thirteen medical educators participated in the study. Three themes were identified from the data that influenced adoption: the wider context (outer setting); perceptions about the innovation; and the medical school (inner context). Participants’ recognition of situations as opportunities or barriers related to their prior experiences of implementing online learning tools. For example, participants with experience of teaching using online tools viewed limited face-to-face placements as opportunities to introduce innovations using virtual patients. Beliefs that virtual patients may not mirror real-life consultations and perceptions of a lack of evidence for them could be barriers to adoption. Adoption was also influenced by the implementation climate of the setting, including positioning of CR in curricula; relationships between faculty, particularly where faculty were dispersed.

**Conclusions:**

By adapting an implementation framework for health services, we were able to identify features of educators, teaching processes and medical schools that may determine the adoption of teaching innovations using virtual patients. These include access to face-to-face teaching opportunities, positioning of clinical reasoning in the curriculum, relationship between educators and institutions and decision-making processes. Framing virtual patient learning tools as additional rather than as a replacement for face-to-face teaching could reduce resistance. Our adapted framework from healthcare implementation science may be useful in future studies of implementation in medical education.

**Supplementary Information:**

The online version contains supplementary material available at 10.1186/s12909-023-04422-x.

## Background

Clinical reasoning (CR) generally refers to the thought processes required to identify likely diagnoses, formulate appropriate questions and reach clinical decisions [1]. In the UK, CR capability is expected of graduating medical students [2]. However, in many instances it has not been explicitly taught in medical schools but rather assumed that students develop their CR skills by knowledge accumulation and observing consultations [3].

Traditionally. face-to-face interaction with real patients has been required in order for delivery of effective CR teaching methods [4]. Students may have limited opportunities for exposure to face-to-face patients, particularly in primary care [5] a clinical setting where CR skills to maximise prompt diagnosis are essential [6]. Teaching through supervised face-to-face consultations also has limitations as students rarely have the time to reflect on their decisions [7].

Teaching innovations using virtual patients can circumvent logistical difficulties in gaining access to real patients, face-to-face [8]. The term “virtual patients” has been used in several different ways. We are using it in its most common form of interactive patient scenarios, where a multimedia presentation of a patient case is used primarily to teach clinical reasoning skills. In learning tools using virtual patients in this form, students typically take on the role of the clinician in a simulated consultation, gather data and make diagnostic and therapeutic decisions [9]. Virtual patient learning tools have the potential to help students improve how they deal with real patients in their practice by offering opportunities for learning by repetition, giving them time to justify their decisions and making the best clinical decisions based on acquired evidence [10, 11], in a safe environment that can be remotely accessed [12]. Adoption of virtual patient learning tools can promote self-regulated learning environments that promote autonomy in learning activities and settings and increase engagement and motivation [13]. Previous research has identified teaching methods that could be most suitable for teaching the different elements of CR skills [14, 15]. Virtual patients have been recognized as particularly useful in improving knowledge organization by providing a varied body of examples of clinical presentations of illness [14]. They are also thought to improve cognitive processes by improving the ability to identify relevant features of a case and interpret clinical information to generate and test hypotheses. Moreover, they do so by providing a more agile learning environment with possibilities of repetition and targeting of complex cases.

There is ample evidence of the effectiveness of learning tools using virtual patients to complement or replace elements of face-to-face teaching in general but also in particular for teaching CR [8]. However, as McGaghie et al. observed in 2016 “integration of simulation into existing curricula is challenging” [16]. They proposed as a research priority the need for increased attention to implementation science, taking account of social processes and context. The context influences not just how implementation takes place but also the extent to which learning outcomes from virtual patients are achieved [17]. Our own research developing and evaluating a novel learning tool using virtual patients called eCREST for teaching CR suggested that such tools can help students improve their reasoning skills [18–20]. Our discussions with medical schools to explore trialling eCREST suggested significant variations in capacity and readiness to adopt and integrate educational innovations. In our experience, where a new resource into the curriculum was more integrated with the curriculum, not only student uptake was higher, but satisfaction was also higher [21].

In this paper, we adapt two implementation science frameworks with an aim to understand from medical educators' perspectives what influences the adoption of virtual patient learning innovations to teach CR in medical schools. We focus mainly on adoption, briefly defined as the initial decision to try an innovation [22–24] which is a determinant of full implementation. Therefore, our research question is: what makes adoption for CR teaching more likely? To answer this, we conducted a qualitative study focusing on medical educators’ perspectives on:

How does the context of medical schools influence the likelihood that virtual patient learning innovations for CR will be adopted?

How do perceptions about two key aspects of the innovation – CR and virtual patients—influence decisions of adoption?

In addition, by adapting implementation science frameworks in a medical education context we offer lessons learnt and how these frameworks can be best used in future research.

## Methods

### Study design and setting

A qualitative study using semi-structured telephone interviews was undertaken. The study was approved by the UCL Research Ethics Committee (reference: 13,497/001).

### Recruitment

We recruited UK medical educators with control over teaching materials and leading CR teaching. Participants were purposively sampled to obtain a range of demographic characteristics across a broad geography. Medical educators were initially identified through the research team’s network and the UK CR in Medical Education Group (CReME). Then, snowball sampling followed, identifying individuals through participants’ professional networks. Approximately twelve to fifteen participants were initially considered as sufficient to reach data saturation according to the topic [25] and this was further evaluated during analyses to decide whether further participants were needed. One author (APK) emailed potential participants to arrange an interview date and obtained written informed consent in advance.

### Theoretical framework

The Unified Theory of Acceptance and Use of Technology (UTAUT) was used to inform the development of the topic guide (see Additional file 1: Appendix I) focusing on four areas that can constitute barriers to individual user adoption: performance expectancy, effort expectancy, social influence and facilitating conditions. The UTAUT is centered on explaining user intentions to use an information system and is thus used to explain subsequent usage behaviour when technology innovations are introduced in organizations [26, 27].

We started from the perspective that educators’ individual intentions would be key for adoption which is why we selected UTAUT to inform the topic guide, but the data suggested otherwise, i.e. that organisational context as also a key determinant. This led us to look at implementation frameworks in which context is typically considered, but we couldn’t identify any developed specifically for medical education. We selected the Consolidated Framework for Implementation Research (CFIR), a conceptual framework that was developed to guide systematic assessment of multilevel implementation contexts to identify factors that might influence intervention implementation and effectiveness. We considered CFIR suitable for our purposes because it is relatively generic and because it comprehensively considers the context of implementation [28–30]. Also CFIR is considered a determinants framework, i.e., used to help understanding what influences implementation [31]. In Table 1 we present the CFIR and UTAUT domains and constructs. In Table 2 we show how the constructs of the CFIR and UTAUT were adapted for use in medical education implementation research, informed by our data. During this mapping exercise, the four subconstructs of UTAUT were mapped on to the CFIR subconstructs relevant to this study to develop the study-specific constructs. *Theme I* captures conditions for adoption in the wider context, e.g. national policies, guidelines and incentives. In CFIR, this is referred to as the “outer setting”. It also includes knowledge of patient needs and resources, which we adapted to medical education as “knowledge of students’ needs and resources”. The inner setting refers to adoption conditions that reflect aspects of institutional needs and resources. In this context, this refers to medical school needs and resources. *Theme II* captures features of the innovation (relative advantage, adaptability, trialability and complexity, which features in both CFIR and UTAUT) as perceived by educators. We use this theme to explore educators’ beliefs and attitudes and identification within the organization, thus combining it with CFIR subconstructs of characteristics of individuals (knowledge and beliefs about the intervention). *Theme III* captures the institutional context. In CFIR this is referred to as the “inner setting”. Subthemes from our data about the medical school context mapped well to the CFIR inner setting subdomains such as “Structural Characteristics”, “Implementation Climate”, and “Readiness for Implementation”. There were few corresponding concepts in the UTUAT for this theme.

**Table 1:** Description of the CFIR and UTAUT frameworks

	**Domains**	**Construct**
**Consolidated Framework for Implementation Research (CFIR)**	Intervention characteristics	Intervention SourceEvidence Strength and Quality Relative Advantage Adaptability Trialability Complexity Design Quality and Packaging Cost
	Outer setting	Patient Needs and ResourcesCosmopolitanism Peer Pressure External Policy and Incentives
	Inner setting	Structural CharacteristicsNetworks and Communications Culture Implementation Climate Readiness for Implementation
	Characteristics of individuals	Knowledge and Beliefs About the InterventionSelf-Efficacy Individual Stage of Change Individual Identification With Organization Other Personal Attributes
	Process of implementation	PlanningEngaging Executing Reflecting and Evaluating
**The Unified Theory of Acceptance and Use of Technology (UTAUT)**	Performance expectancy	The degree to which an individual believes that using the system will help him or her to attain gains in job performance
	Effort expectancy	The degree of ease associated with the use of the system
	Social influence	The degree to which an individual perceives that important others believe he or she should use the new system
	Facilitating conditions	The degree to which an individual believes that an or’anisation's and technical infrastructure exists to support the use of the system
		Moderators: age, gender, experience and voluntariness of use

**Table 2:** Adapting CFIR and UTAUT Frameworks for Medical Education Implementation of virtual patient simulation tools

Study sub-themes/adapted construct	Short description	Related CFIR Construct(s)	Related UTAUT Construct(s)	Examples of evidence in this study	Facilitator or Barrier in the context of this study
**Theme I: External context: influences of adoption (CFIR “Outer setting”)**					
Limited opportunities for learning CR through interactions with real patients	The extent to which student needs, as well as barriers and facilitators to meet those needs influence need for adoption	External Policy & Incentives Patient needs & resources	None	Tutors reported that increasing numbers of students led to limited placements available, constraining opportunities for face-to-face interactions with real patients	Facilitator
Knowledge of students’ needs and resources: previous experience with online learning	The extent to which students’ previous experience with online learning influence their future acceptability	Patient needs & resources	Experience, Social Influence	Tutors reported that student’s diverse familiarity with online teaching can impact their readiness to accept	Facilitator if previous experience is positive
**Theme II: The features of the innovation**					
Perceived benefits and challenges of using virtual patients as educational tools – providing the evidence	Stakeholders’ perception of the quality and validity of evidence supporting the belief that the intervention will have desired outcomes	Evidence Strength & Quality, Knowledge and Beliefs about the Intervention	Evidence strength/quality	Tutors reported that often lack of evidence on available resources constitutes a barrier to adoption	Facilitator if evidence can be provided
Perceived benefits and challenges of using virtual patients as educational tools – providing the evidence	Stakeholders’ beliefs about the intervention	Relative Advantage, Knowledge & Beliefs about the Intervention (follows from themes I-II)	Knowledge and beliefs about the resource, Performance Expectancy, Effort Expectancy, Other personal attributes (motivation), Other personal attributes (values), Relative advantage	Tutor beliefs about advantages and disadvantages of virtual patients	Facilitator if the advantages outweigh the disadvantages
Perceived benefits and challenges of using virtual patients as educational tools – providing the evidence	**Experience with online teaching**: The extent to which the stakeholders’ previous experience with online learning influence their adaptation	Individual Stage of Change	Experience, Self-efficacy, Other personal attributes (motivation)	We found that tutors’ own diverse familiarity with online teaching can impact their readiness to adopt	Facilitator if the experience is positive
*Beliefs about* using virtual patients as educational tools—distinction between reality and simulation	**Distinction between reality and simulation**: The degree that the intervention is realistic in terms of the online patient simulations	Knowledge & Beliefs about the Intervention	Knowledge and beliefs about the resource, Other personal attributes (motivation), Other personal attributes (values), Relative advantage	Tutors reported that virtual patients should mirror how students should consult with patients in real life	Facilitator if the resource is realistic
*Beliefs about* using virtual patients as educational tools—distinction between reality and simulation	**Distinction between linearity and complexity**: The degree that the intervention is realistic in terms of the patient scenarios	Knowledge & Beliefs about the Intervention	Knowledge and beliefs about the resource, Other personal attributes (motivation), Other personal attributes (values), Relative advantage	Tutors reported that virtual patients should mirror the complexity of real-life consultations with patients (including question-orientation)	Facilitator if the resource is realistic
**Theme III: Institutional context: opportunities and barriers for adoption (CFIR “inner setting”)**					
Explicit positioning of CR in curricula	**Receptiveness of change (materials):** The extent to which the nature and communication of teaching material enable curricula change	Implementation Climate, Readiness for Implementation	None	Tutors reported that the diversity of teaching CR with different terms constitutes a barrier to adoption and reinforces uncertainty	Barrier
Explicit positioning of CR in curricula	**Receptiveness of change (CR as a subject):** The extent to which CR current teaching enables curricula change	Implementation Climate, Readiness for Implementation	None	Tutors reported that the lack of explicit positioning of CR in curricula constitutes a barrier to adoption (CR not explicit)	Barrier
Explicit positioning of CR in curricula	**Feasibility of change:** Stakeholders’ perception of how feasible is to introduce a change in medical school curriculum	Structural Characteristics, Implementation Climate, Relative Advantage	None	Tutor reported that medical school curricula are inflexible to changes that makes introducing a new resource challenging	Barrier
Decision-making for adoption	Norms, processes and basic assumptions of a medical school	Structural Characteristics, Culture, Implementation Climate	None	Tutors reported that decision-making process and integration in medical schools depends on scale	Facilitator if introduced on a small scale first
Decision-making for adoption	**Dispersed faculty:** The positioning of internal networking requirements for adoption	Networks & Communications, Access to Knowledge and Information, Individual Identification with Organization	Social Influence, Facilitator Conditions	Tutors reported that other adopters can help facilitating adoption	Facilitator
Decision-making for adoption	**Positioning of intervention:** The degree that additional interventions fit with existing teaching practices and how these can be combined	Implementation Climate, Knowledge & Beliefs about the Intervention, Relative Advantage, Compatibility	None	Tutors reported that positioning of virtual patients as additional instead of replacement can bend resistances to adoption	Facilitator
Decision-making for adoption	**Identification with institution:** The extent to which individuals identify themselves with the medical school, and their relationship and degree of commitment with the school	Individual Identification with Organization	Individual identification with organization, Other personal attributes (motivation)	Tutors reported that the institutional model of using NHS doctors to deliver teaching means they don’t have institutional levers to enforce changes	Barrier

### Data collection

Telephone interviews were conducted between October 2019 and February 2020 by one author until data saturation was reached. The topic guide was piloted among the research team and with two medical educators as experts to inform analysis. Interviews were audio-recorded and transcribed verbatim via a professional service which did not have access to any identifiable information. As part of ensuring interviewer reflexivity, we disclosed the research team’s involvement with an online tool using virtual patients [18–20] and ensured that all information provided were anonymous and not to be shared with the interviewee’s institution to build trust and an honest conversation.

### Data analyses

A thematic analysis approach using a combination of deductive analysis based on the interview topic guide and inductive coding of transcripts [32] was undertaken. One author (APK) systematically searched for patterns within participants’ reflections and analyzed the textual transcribed data by reading text, then creating unfocused, descriptive, conceptual, and linguistic notes, generating codes and considering themes or clusters of themes. The deductive analysis based on mapping the codes into the CFIR subconstructs of relevance began soon after the start of data collection, and field notes taken by the researcher during the interview and analyses were used to reflect on previous responses during interviews. The inductive analysis was then used in order to consider the participant reflections within the context of implementation research and innovation adoption. Therefore, themes identified in one interview were explored in subsequent interviews to ensure the robustness of thematic analysis, and that coding was informed iteratively by accumulating data and ongoing analyses.

Two steps of reliability checks were used to ensure robustness of the analysis process. The first three transcripts were double coded by a second author (JS) who generated codes to ensure that all possible themes have been detected and to improve the reliability of the analysis. These were discussed in a meeting between the authors. The second step involved a third author (RP) checking the application of the coding of the first ten transcripts and a meeting exploring additional codes.

## Results

### Sample

The final sample comprised thirteen medical educators with 16 median years of experience (SD = 5.6) and 13 median years (SD = 6.5) at the institution they were employed when interviewed. The majority were female (62%) and they worked at institutions covering a range of areas in England, Scotland and Wales. Almost half (*n* = 6) had experience with introducing some type of online or blended innovations for teaching CR, including virtual patients, at the time they were interviewed (Table 3).

**Table 3:** Information about participants in the study (*N* = 13)

Participant	Age	Gender	Area	Years of experience	Years at institution	Experience of using online innovations for teaching CR
Participant A	34	F	Norfolk	7	7	Yes
Participant B	NR	F	South Wales	NR	NR	No
Participant C	57	F	Eastern Scotland	15	2	No
Participant D	37	M	Western Scotland	8	3	No
Participant E	54	M	East Midlands	14	14	Yes
Participant F	56	M	London	25	21	No
Participant G	45	F	London	13	13	Yes
Participant H	50	M	East Midlands	20	7	Yes
Participant I	58	F	North East England	20	16	No
Participant J	48	F	North East England	18	16	Yes
Participant K	51	M	East of England	20	15	Yes
Participant L	36	F	West Lancashire	8	1	No
Participant M	59	F	West Midlands	17	13	No

### Application of theoretical framework

#### Main themes

**Fig. 1:**
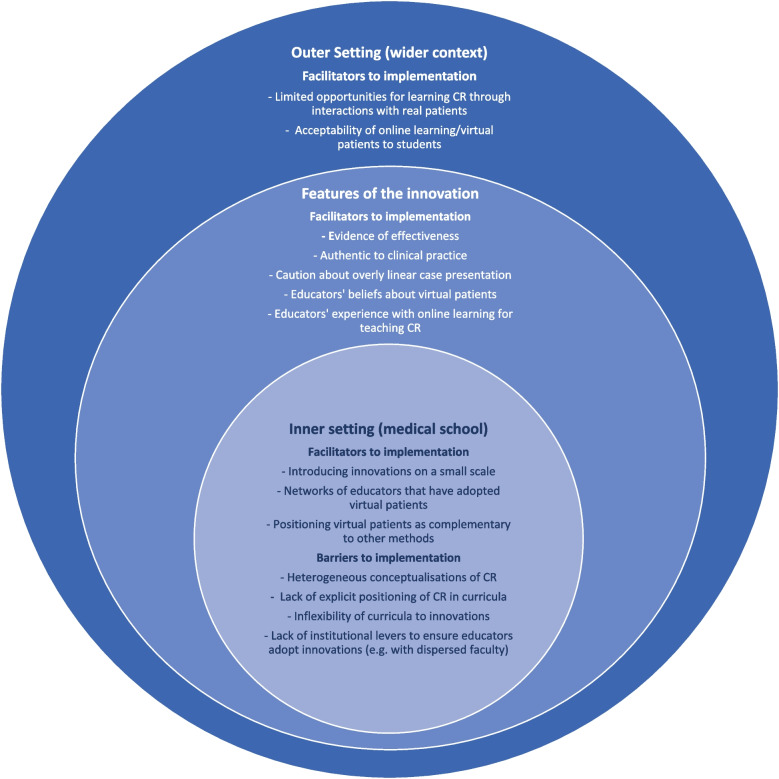
Conceptual framework for the implementation of virtual patient simulation tools in medical education based on the perceptions of educators

Participants’ accounts fell under three themes and six sub-themes related to conditions influencing adoption of learning tool using virtual patients, described in Table 2. Those related to the outer setting influences of adoption (theme I) included the limited opportunities students have for learning CR with real patients and students’ previous experience with online resources. Those related to the features of the innovation (theme II) included both teaching of CR in general and the application of virtual patients to deliver it. Finally, the inner setting opportunities for adoption (theme III) related to decision-making processes for adoption, the educators’ perceived benefits of using virtual patients for teaching CR and their identification with their institution. Figure 1 provides an overview of the conceptual framework derived from educators’ perceptions of the barriers and facilitators to implementation of virtual patient learning innovations in medical education in this study.

We also found that the participants’ views on what constituted a barrier or an opportunity for adoption differed by their experience in introducing online learning tools for teaching CR and, therefore, we include this information with illustrative quotes. We now describe the three themes using examples of quotations from participants’ accounts to support them.

### Theme I: Wider contextual influences of adoption

#### SMBE presented a learning opportunity when interaction with real patients was limited

Several medical educators considered that online simulation-based tools using virtual patients may help them circumvent the sparsity of placements in a wide range of different places or at different times of the year and reduce variation that usually occurs in face-to-face case-based learning.

*...the pluses are that you can engage with virtual patients that you potentially would find difficult to get to come into a placement setting, either due to geographical issues or their mobility issues or it might be a group of hard-to-reach patients that it’s hard to engage with and get them prior to placement and students meet face-to-face, so I think it addresses that side of things quite well, and it’s a way that you can control to a certain extent what the patient is coming out with.* [Participant A, Experience of teaching CR using online learning tools]

They tended to introduce online learning tools with virtual patients to meet insufficient placements for students, with innovation adoption being opportunistic.

*… In essence we took an opportunistic bit of serendipity where there were insufficient placements (…) And, we covered various things from diagnostic thinking through to errors of biases, information management, sharing in decision-making et cetera.* [Participant E, Experience of teaching CR using online learning tools]

Some medical educators did not have experience of online learning tools using virtual patients, and they associated virtual patients with online consultations.

*So, I’m not saying that you can’t do it online; I’m just thinking, clinical practice at the moment is not very much an online exercise.* [Participant M, No experience of teaching CR using online learning tools]

#### Knowledge of students’ needs and resources

Educators reflected on the implications of students’ prior use of online learning tools in their medical education. In some circumstances, students’ familiarity with previous online learning tools used in their course could be helpful.

*And, when they came to the fourth-year course, they were already armed with those tools, and we could actually get into the ‘nitty gritty’ of using online resources to support your CR.* [Participant E, Experience of teaching CR using online learning tools]

Another medical educator also recognized that students feel a gap and a need for more resources to teach CR.

*I think they would use it. And, you know, especially in terms of … I think they recognise they need to develop their CR.* [Participant B, No experience of teaching CR using online learning tools]

In addition, incorporating online learning into teaching may enable the participation of ‘quieter’ students who may normally not interact with others in a classroom.

*An online package would enable those quieter students to still work through a case, and in the free text boxes, they have to comment, they have to have the voice and say what they want to do.* [Participant L, No experience of teaching CR using online learning tools]

### Theme II: The features of the innovation

#### Perceived benefits and challenges of using virtual patients in educational tools – providing the evidence

For medical educators, it was important that innovation developers provide strong evaluation evidence of the resources they introduced in terms of the resources’ efficacy, usability and feasibility. This may influence their motivation to adopt, or their efficiency in persuading their medical school on the value of introducing a change in the curriculum.

*Have they completed it, time to completion, if there is some sort of post… about pre-knowledge check, post-knowledge check, and evaluation of whether or not they found it useful? […] What are the areas identified that have been problematic and need to be covered off? Evaluation is necessary for medical educators to be convinced on the SBME methods trialability and it has to be embedded early.* [Participant D, No experience of teaching CR using online learning tools]

Educators also mentioned needing innovators to demonstrate how the innovation works; compatibility with existing technology and support in place for updating content according to changes in clinical guidelines.

*I think, obviously, information, and the opportunity to have a demonstration of the innovations … and it’s then how you disseminate it out and who is going to be using it. … and then there’d have to be some sort of support for disseminating it out to other groups. In medical schools where we have dispersed learning, that could be quite tricky geographically. Then there would need to be support and backup, until people were confident and competent to take it forward for themselves.* [Participant C, No experience of teaching CR using online learning tools]

#### *Beliefs about using virtual patients in educational tools –* distinction between reality and simulation

The participants further described their own beliefs and attitudes towards virtual patients. Some discussed the adoption of these tools critically, questioning whether virtual patients reflect how patients act in real life (see Table 4, distinction between reality and simulation).

*That [to control the content of consultations] can be a plus, but it also can be a negative because looking at the way that the questions come up in a formulaic way that the students ask them, they ask them and it follows an algorithm is great but, obviously, patients in reality don’t do that.* [Participant A, Experience of teaching CR using online learning tools]

**Table 4:** What would make virtual patient learning tools more likely to be adopted to teach CR

These are the lessons learnt from this study, which can be seen as additional to having a solid evidence-base of effectiveness and acceptability of learning tools using virtual patients
a) Introducing learning innovations using virtual patients is more likely when CR has been explicitly taught already because both student and tutors have a language to express what they are doing, and therefore learning can be recognized and measured
b) Opportunistic implementation of online learning innovations using virtual patients to address limited face-to-face teaching capacity can build local support for virtual patients and may reduce institutional barriers to formally approving curriculum changes
c) Training and sharing information on key features of learning tools using virtual patients can help, that emphasises: - the position of virtual patients as complementary rather than a replacement of face-to-face teaching - capacity for online learning approaches to involve students that are typically quiet during face-to-face teaching - opportunities for using virtual patients in group learning situations not just individual study - ways in which virtual patient learning tools can save or optimize tutor time
d) Adoption is more likely when evidence of effectiveness and acceptability are combined with support for dissemination, cases and scenarios that are realistic, and with adaptable resources by developers

Adaptability was consistently discussed by medical educators who were sceptical whether online methods can simulate how real-life consultations take place and whether the conceptual linearity often imposed by virtual patient learning tools could reflect the complexity of face-to-face clinical practice (see Table 1, distinction between linearity and complexity).

*...one of the things I think online approaches delivering these things have to try and get past is the inevitable linearity of the way patients present, because in the messy world of face-to-face clinical practice, things don’t come at you in a sequence the way that they are often presented… Whereas actually, in real life, that information is hidden among other things that are absolutely no assistance to me whatsoever when I am trying to make a diagnosis.* [Participant E, Experience of teaching CR using online learning tools]

Moreover, some medical educators perceived VPs as primarily question-oriented, and thus forcing students to ask more questions rather than ask the right questions to ascertain their differential diagnoses.

*Yes, my experience of the online patient simulation is they tend to be very question-orientated and they actually undermine good quality CR in a clinical setting, because in order for the algorithms to work what they end up doing is they push you to ask questions. By asking a question – certainly what I’ve seen of the simulated scenarios – it pushes you through the algorithm and it actually encourages poor consultation skills.* [Participant K, Experience of teaching CR using online learning tools]

On the other hand, other medical educators highlighted that it is the interaction with the patient which is important either with a simulated patient online or an actor or a real patient face-to-face.

*I think using simulators actually can tend to kind of control and regulate some of those other aspects a little bit better and perhaps give some more consistent things. But certainly, I think actually getting them to do the CR, to be talking to patients or simulators is a more effective way I think than actually just teaching them about CR or about conditions.* [Participant H, Experience of teaching CR using online learning tools]

### Theme III: Inner setting opportunities and barriers for adoption

#### Explicit positioning of CR in curricula

Receptiveness of change of schools’ curricula related to both introducing materials (i.e., virtual patients in educational tools) and making changes to CR as a subject (see Table 1). For example, participants reflected on the difficulty of introducing new materials given the position of CR in the school curriculum which is not always secure.

*It’s a little bit more restricted, just because, in general, those kinds of courses are a lot bigger, there are a lot more people involved; there’s a national curriculum, they’re regulated and inspected by the GMC [UK General Medical Council].* [Participant L, No experience of teaching CR using online learning tools]

Medical educators with no experience with simulation-based online learning tools linked the difficulty in convincing their institution to adopt a new method of teaching with wider questions about the value of CR teaching in the curriculum, where they felt the evidence-base was lacking.

*At [University name], I had a few attempts to try to see whether it would be possible to make a more transparent continuing CR pathway for students to be working through, but it doesn’t seem to have been adopted. It’s not something that the school has really embraced. And I think that part of the reticence around that is not being convinced that there is sufficient evidence that being overt and teaching specific approaches for CR leads to improved outcomes for decision-making as doctors.* [Participant F, No experience of teaching CR using online learning tools]

Most participants reflected there are implications of not teaching CR explicitly for both perceived and actual learning. Students are not always conscious that they are taught CR, and this makes it very difficult for them to reflect on what they have learnt and whether they have improved or not.

*My understanding of it is that it isn’t explicit. (…) But I think it’s been called clinical relevance and different names, so I think if you ask the medical students what’s CR, they wouldn’t explicitly know that they’ve been assessed on it, (…), it’s going to be an issue because if they don’t understand that they’re being necessarily assessed on it or what it is, then it’s hard for them to know whether they have improved or what’s going on with it…* [Participant A, Experience of teaching CR using online learning tools]

In addition, one medical educator noted that both students and teachers are missing the vocabulary that is necessary to understand CR. This makes it difficult for students’ reflection to take place and there is no capacity for students to observe how teaching is helping them.

*I would say probably up until the last few years, [students] weren’t taught the reasoning overtly, therefore, they weren’t taught the vocabulary. So, to talk about inductive reasoning or hypothetical deductive reasoning, or type one and type two thinking, or to talk about better cognition or cognitive forcing strategies. Those words just aren’t there for them, so in order to have a conversation about something, you need to have words that everybody understands. Those words obviously need to be underpinned by a knowledge of the concept. Without that, it’s very, very difficult to guide reflection if you don’t have those words.* [Participant C, No experience of teaching CR using online learning tools]

Medical educators observed that this tension of CR is manifest also in assessment.

*Because I think the whole thing about CR is that often, real life patients don’t fit into the boxes with some of the vague symptoms that don’t really fit into any one category, and you could go down any direction just to find the answer. And, sometimes, you extensively investigate a patient, and never quite get to the bottom of what’s wrong. And we don’t like that in education, because we like to write […] questions that are very binary almost; like, right and wrong, and students like the right and wrong, as well. So, I think sometimes there’s not always enthusiasm for CR.* [Participant L, No experience of teaching CR using online learning tools]

#### Decision-making for adoption

On an organizational level, medical educators believe online learning innovations in general are more likely to be accepted in a stepwise way to avoid large-scale changes.

*I think the institution is very interested in innovation and development, but it depends a little bit on the scale, if it’s a small change in the small part of the curriculum, then it’s obviously much easier than if it’s some big scale curriculum wide change.* [Participant C, No experience of teaching CR using online learning tools]

A more experienced medical educator with simulation-based online learning tools considered that other conditions necessary for adoption include the expected effort as well as the social influence from others that can impact their motivation to adopt.

*Quite a lot of effort, really, to try and get anything new into a curriculum in a medical school is quite difficult, although this is good because … it moves into blended learning and putting things online and I know there’s definitely a movement to do that.* [Participant A, Experienced with simulation-based online learning tools]

Medical educators also preferred when online learning tools were introduced as a supplement to traditional methods rather than as a replacement.

*I would just say, “It’s part of the menu”. I would be clear, I think, about not replacing… It’s not replacing anything that’s done already; and I think perhaps what it does, it gives the chance of the students to have a go.* [Participant I, No experience of teaching CR using online learning tools]

One participant suggested that a blended learning method can motivate medical educators in terms of minimizing and not increasing their effort because they could use the time students will spend with online teaching towards their own clinical work.

*And I suppose you might sort of say, “Well, that could be a benefit to the tutor, in that the students might do the online resource for an hour where you could see some patients; then they need to have a tutorial, and then they would do the parallels”. (…) So, I suppose selling it to faculty, it could be that this is the way of… You know, when the tutor is busy, or out of actual one-to-one teaching time, that the students are still doing very relevant clinical work.* [Participant I, No experience of teaching CR using online learning tools].

Some medical educators found it difficult to introduce innovations in the curricula when the teachers are clinicians employed by the NHS where leverage for changing their teaching practice is limited.

*…often, the people who are teaching the students are not employed by the academic institution. […] That causes a huge amount of difficulty in terms of introducing an innovation that involves those teachers because they don’t know – and the communication to them is very poor – about the rationale behind decisions. They’re not very closely involved in decision-making about how things change. They tend to revert to whatever they’ve always done because they’re busy with their NHS jobs and teaching students is something they see as an almost unpaid add-on to their role.* [Participant F, No experience of teaching CR using online learning tools].

## Discussion

### Main findings

This qualitative study has elucidated the interactions between the context and adoption decisions regarding online learning tools using virtual patients in teaching CR (see Table 4). When adopting new teaching methods, the focus is usually on the characteristics of an intervention [33], but this study shows that the features of the setting and the individual’s relationship to it are also important.

### Comparison with other studies and framework of implementation

McGaghie et al. [28] has previously highlighted that implementation difficulties could limit the potential of simulation-based online learning tools such as those using virtual patients, and suggested that focusing on implementation science is one of four key areas for advancing implementation of innovations in teaching using simulations [16]. Studies like this provide further guidance on theories, tools, resources and outcomes that need to be considered when reporting implementation findings in medical education [34]. This is important because if virtual patient innovations are well implemented they have the potential of yielding mean effect sizes almost three times higher than poorly implemented ones [35]. As a recent editorial highlighted, the effort is to increase implementation capacity in simulation-based medical education ‘by offering a systematic approach to program implementation’ [17]. This is in turn may increase students’ positive engagement with new learning tools, which is crucial for wider adoption [36–39].

### Strengths and limitations

To the best of our knowledge, this study is the first to explore medical educators’ perceptions on adoption of virtual patient learning innovations for teaching CR. It is also the first to our knowledge to adapt an implementation framework to a medical education context.

CFIR is designed to inform evaluation of an implementation strategy as a determinant framework explaining influences of implementation outcomes. [40], We adapted CFIR in order to understand what factors might influence the implementation of virtual patient learning tools, with no specific implementation strategies under study. While CFIR was useful on considering the context of adoption, we also found that some parts overlapped with UTAUT. In some cases, this could be a feature of our study design since all our data came from interviews, therefore individual perceptions and attributes of the innovation overlapped because attributes were drawn from individual perceptions. A larger case study approach with more data sources (e.g., observations, documents describing the innovation from more perspectives) could enable these concepts to be better separated.

Our adapted framework enabled examination of context and social processes influencing adoption and can be used for other medical education innovations beyond targeting only individual learners’ competence, knowledge and performance [41]. It may not be applicable without further adaptation to studies seeking to understand embedding of innovations already adopted, or the design of implementation strategies.

Our study had a UK sample size (*n* = 13) which may limit the extent to which our findings can inform medical education outside the UK. However, clinical reasoning is taught beyond the UK and the role of virtual patient learning tools in medical education is growing in countries outside the UK such as the US and other European countries. Some of the medical educators that participated in the study were already involved in developing simulation-based online learning tools which may have provided them with a broader perspective than those that had little experience of these tools. By exploring the views of those with and those without experience of teaching using these tools we captured some differences in practice and in training. This experience with simulation-based online learning tools provided valuable insights into barriers and facilitators of adopting these tools from personal experience. Also, we are aware that medical educators’ perceptions of their students’ experience with simulation-based online learning tools may not reflect students’ actual experiences. However, this study provided valuable insights on educators’ perceptions which are valuable in designing appropriate implementation strategies. Finally, the interviewer and other authors involved in interpreting the data were also involved in developing a virtual patient learning tool [18–20].

### Implications for medical education and future research

Since this study was conducted, the context of simulation-based online learning tools in medical schools has changed further due in large part to the normalization of online technology in medical education following COVID-19 [42]. Also, there were several wider contextual changes in medical education and clinical care [43–45] that are leading to greater opportunities to use simulation-based online learning tools in medical education. The experience of teaching during the COVID 19 pandemic and threats of future pandemics placing greater reliance on online teaching methods [46, 47]; the normalization of online medical care, may reduce the gap between online learning and knowledge in medicine. The removal of the cap on the number of medical school places will lead to further increases in medical school student numbers, placing greater pressure on the availability of face-to-face learning opportunities [48, 49].

In this context, the study findings have implications for medical educators considering adopting simulation-based online learning tools using virtual patients and innovation developers. For medical educators who need to navigate organisational challenges to implementing virtual patient learning tools, the following points may be helpful. Organisational support for blended learning and virtual patient introduction is important. To encourage organisational support educators could stress how using virtual patient learning tools as part of a blended learning approach may address the pressures experienced by medical educators by reducing work load in developing/delivering teaching, and facilitating more standardised delivery across educators. Emphasizing other benefits of virtual patients in online learning is also important such as it can lead to greater inclusivity and student engagement, particularly with quieter students, and fills a significant gap in the curriculum in the teaching of explicit CR skills. Similarly, it may be helpful to be aware that varying conceptualisations of CR exist, both across and within Health Professions education [8, 50]. For example, a scoping review mapping clinical reasoning literature identified six different categories of terminology used across Health Professions education that capture the different elements of clinical reasoning: skills, performance, process, outcome, context, and purpose/goal [51]. These may explain some of the preconceptions held by educators and others about how virtual patient learning tools can teach CR skills, such as their reservations about the simplification/linearity of virtual patient scenarios and the fidelity to clinical situations. For example, fidelity can be important for specific skills but high fidelity is not always superior to lower-fidelity as it depends on what is taught and the learners’ level of knowledge [52]. Virtual patients are potentially a useful teaching method for improving specific CR skills such as knowledge organisation and cognitive processes [14]. For teaching these elements of CR, the fidelity and linearity of the cases may be less important but being exposed to several varied cases where the object is to identify features and generate and test hypotheses is valuable. Virtual patients are, therefore, complementary to other methods of teaching CR skills that may focus on other elements [8]. Additionally, for novices learning in a simulated reality, a departure from a complex reality can have benefits as it removes other factors that can interfere with the learning of a specific skill [16]. Educators should consider which elements of CR they are currently teaching and how, to understand the value of complementary tools like virtual patients and what value they can add to their curriculum and pedagogic methods.

For innovators, as expected, educators need evidence of effectiveness and user acceptability. They also need support for dissemination to a range of other educators, cases and scenarios that resemble realities in clinical practice, and with resources that are adaptable in terms of content, feedback and to learner needs [53]. Characteristics like embedded feedback, opportunities for reflection, consistency of learning experience are important because the deliberate and active engagement of students is necessary to learn [54]. This was highlighted by one participant who felt that virtual patient simulated consultations can be too question orientated, which hindered reflection and worsened CR skills. Therefore, further research is needed, on understanding what educational providers need and what needs to be in place across different institutions to ease adoption and implementation. This is crucial in times when the NHS is being transformed to provide new models of care using workforce other than medical professionals to prescribe such as pharmacists and physician associates [55].

## Conclusions

This exploratory study focused on circumstances that can facilitate adoption of simulation-based online learning tools using virtual patients for teaching clinical reasoning. By adapting the CFIR, we were able to identify features of current teaching processes and the implementation climate of medical schools that seem important in the adoption of virtual patient learning tools. These include access to face-to-face teaching opportunities, positioning of clinical reasoning in the curriculum, relationship between educators and institutions and decision-making processes.

Our adapted framework may inform future studies by indicating the variables that could be examined quantitatively in assessing readiness for implementation in institutions and amongst educators. It could provide a framework that could be further adapted for analysis of a larger scale qualitative exploration of implementation of virtual patient innovations.

## Supplementary Information

**Additional file 1:** **Appendix I.** The interview topic guide used in the study.

## Data Availability

The datasets used and/or analysed during the current study are available from the corresponding author on reasonable request.

## Abbreviations

GP: General Practitioners
SBME: Simulation-Based Medical Education
NHS: National Health System
COVID-19: Coronavirus disease
eCREST: Electronic Clinical Reasoning Educational Simulation Tool
CFIR: Consolidated Framework for Implementation Research
CREME: UK Clinical Reasoning in Medical Education Group
GMC: General Medical Council
NVQs: National Vocational Qualifications
OSCE: Objective Structured Clinical Examination
ERIC: Expert Recommendations for Implementing Change
