# Supplementary material for: Educators’ perspectives of adopting virtual patient online learning tools to teach clinical reasoning in medical schools: a qualitative study
Source: BMC Med Educ. 2023 Jun 8;23:424. doi: 10.1186/s12909-023-04422-x (PMC10248983; doi:10.1186/s12909-023-04422-x)
Supplement: Supplementary file 1 — Additional file 1: Appendix I. The interview topic guide used in the study. [file 12909_2023_4422_MOESM1_ESM.docx]

Appendix I: The interview topic guide used in the study

Aim: To explore barriers and facilitators of adopting virtual patient methods to teach clinical reasoning in medical schools

Plan: 30-60 minute telephone interviews

1. Current practice (personal experience)

Can you let me know about your experience of teaching clinical reasoning?

What approaches have you found more effective/useful?

1. Current practice (institution)

Can you let me know how clinical reasoning is taught at your institution? [prompt: do you use any online/blended methods?]

[if online/blended methods are used) What is your experience with these methods/approaches? Are they effective in improving students’ clinical reasoning?

[if no online/blended methods are used) What is your experience with these methods/approaches? Are they effective in improving students’ clinical reasoning? Did you or your institution consider introducing online methods? Why not/What happened?

Which factors you think affect sustainable adoption of new methods? How important is integration of any new method/tool in the curriculum in terms of adoption?

1. Individual factors for adopting innovation

[performance expectancy] What do you think you can gain and lose/risk as a professional from introducing online and/or blended methods of teaching clinical reasoning?

[performance expectancy] What do you think your students can gain and lose/risk from introducing online and/or blended methods of teaching clinical reasoning?

[effort expectancy] How does developing and delivering simulation-based methods fit with your work role at your university? How much effort would that require from you and other members of staff?

[effort expectancy] How much effort would you be able to perform to adopt new teaching methods?

[effort expectancy] How will the introduction of online or blended methods affect your performance with other institutional duties?

[social influence] Who at your institution decides the content/methods of teaching (clinical reasoning)?

[social influence] What is your Institution’s attitude towards institutional changes/introduction of innovation?

[social influence] What is your colleagues’ attitude towards institutional changes/introduction of innovation?

[social influence] How important are the attitudes of senior staff at your Institution on adopting innovation?

[facilitating conditions] Are there any processes/guidelines in place at your Institution to facilitate the adoption and implementation of an innovation?

[facilitating conditions] What would you expect from senior staff at your Institution to help with the implementation of an innovation?

1. Characteristics of the innovation

If a new method is introduced at your Institution, what elements would you expect to see? Some examples for prompts: Feedback elements, Deliberate practice, Curriculum integration, Outcome measurement Simulation fidelity, Skill acquisition/maintenance, Mastery learning, Transfer of practice, Team training, High-stakes testing, Instructor training, Educational/professional context.

What would you expect from the innovation developers to help with the adoption of the innovation?
